# Competitive Size Effects in Antiferromagnetic|Ferrimagnetic Core|Shell Nanoparticles for Large Exchange Bias

**DOI:** 10.1021/acsanm.4c05505

**Published:** 2024-12-04

**Authors:** Alberto López-Ortega, Beatrice Muzzi, Cesar de Julián Fernández, Claudio Sangregorio

**Affiliations:** † Departamento de Ciencias, Universidad Pública de Navarra, Pamplona E-31006, Spain; ‡ Institute for Advanced Materials and Mathematics (INAMAT^2^), Universidad Pública de Navarra, Pamplona E-31006, Spain; § 201843 ICCOM - CNR, Sesto Fiorentino (FI) I-50019, Italy; ∥ IMEM - CNR, Parma I-43124, Italy; ⊥ Dept. of Chemistry “U. Schiff”, University of Florence and INSTM, Sesto Fiorentino (FI) I-50019, Italy

**Keywords:** nanoparticles, core|shell, exchange bias, antiferromagnet, blocking temperature

## Abstract

A family of exchange-coupled core–shell (CS) nanoparticles composed of an antiferromagnetic (AFM) core (Co_0.3_Fe_0.7_O) and a ferrimagnetic (FiM) shell (Co_0.6_Fe_2.4_O_4_) was investigated to unravel the role played by the dimension of the two components on the magnetic properties of the system. The series comprises three samples with different core diameters (2, 5, and 16 nm) and fixed shell thickness of ∼2 nm. Although a strong core and shell magnetic coupling occurs in all the samples, the final properties of the hybrid nanosystems are greatly influenced by the size of the two counterparts. Indeed, while the larger sample can be described as a classic *T*_C_ > *T*_N_ exchange-bias, where *T*_C_ and *T*_N_ denote the ordering temperature of the FiM and AFM phases, respectively, on reducing the size, the blocking transition of the FiM shell decreases to values well below the *T*_N_ of the AFM. In the first case, the FiM-AFM exchange-bias effect is determined by the magnetic ordering of the AFM core; in the other cases, it is due to the reduction of the thermal-driven magnetic fluctuations of the ordered FiM shell. On the other hand, the AFM properties of the core regions also are extremely sensitive to the particle size reduction, showing, for the smallest sample, the effect of the coupling between the two phases to appear at temperature well below *T*_N_ displayed by the bulk system, indicating the potential presence of a blocking transition in the AFM core for small particles. These findings highlight the significant influence of the size of the AFM and FiM components on the hybrid system’s ultimate properties. This result is potentially relevant for defining the working conditions of nanodevices exploiting exchange-bias phenomena, which have been recently proposed in the literature for application in several technological fields, ranging from rare-earth free magnets, spintronics, optoelectronics, and magnetic-refrigeration.

## Graphical abstract

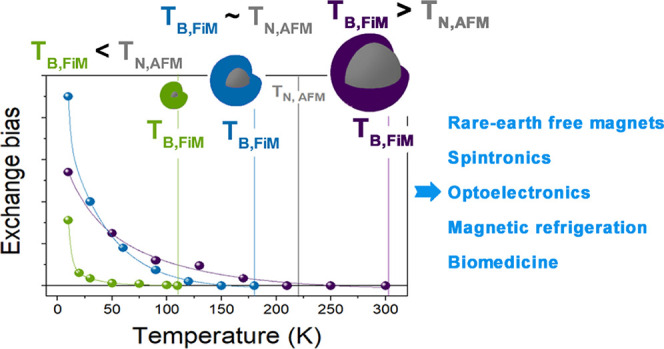

## Introduction

The development of novel multifunctional core|shell (CS) nanoparticles (NPs) has become increasingly appealing in recent years, with the aim of engineering novel multicomponent materials with peculiar physicochemical properties.^1,2^ This research, in parallel with the improvement of the synthesis^3^ and fabrication^4^ methodologies of NPs, has paved the way toward unprecedented multifunctional systems with unique properties.^5,6^ Finite-size and morphological size-dependent properties are critical aspects to consider when developing the desired functionality of the final CS structures.^7,8^ The full exploitation of such effects relies on the ability to independently vary every single parameter involved, as well as of fine controlling the interface shared by the coupled materials.^9,10^

Among the others, particular attention has been paid to nanosystems in which one or both components are made of magnetic materials. Polymers^11^ and organic^12^ or inorganic nanomaterials,^2,13^ among others, have been extensively combined with magnetic structures seeking for novel applications. However, assembling materials with different magnetic properties in CS entities is probably the most known nanoheterostructures endowed with magnetic functionalities.^14,15^ Ferromagnetic (FM), ferrimagnetic (FiM), and antiferromagnetic (AFM) materials have been extensively combined to tune the final magnetic properties of the nanoheterostructure by means of exchange-coupling phenomenon.^14,16^ Spring magnets^15^ and exchange bias^14^ systems are examples of these types of hybrid nanostructures. While the first ones are made up of combinations of FM and FiM structures with different magnetic anisotropy, the second ones exhibit an extra magnetic anisotropy triggered by the coupling of the FM or FiM material with an ordered AFM structure.^17^ Both behaviors can be observed when the size of the material is reduced to the nanometric range. They have been extensively studied in the literature, and their origin is well-known to be related to the coupling of the spins located at the interface of the two materials.^14^ However, other aspects also play a crucial role in the final magnetic properties of these exchange-coupled systems, such as the size and morphology of each component and the peculiarity of the CS architecture.^18^ Moreover, regarding the exchange-bias in CS nanostructures, the relative value of the Curie, *T*_C_, and Néel, *T*_N_, ordering temperatures of the two components can also induce peculiar magnetic feature in the hybrid structure.^19,20^ In this context, it should also be expected that thermal fluctuations, which in single-domain particles can force the transition of the magnetic material from a blocked to a superparamagnetic state,^21^ can play a role. However, the effect of this extra degree of freedom has been scarcely considered so far.

Here, we present an investigation aimed at filling the gap by exploring the specific role played by each magnetic component in a series of AFM/FiM exchange-coupled CS NP. The system is composed of a single inverted AFM­(Co_0.3_Fe_0.7_O)|FiM­(Co_0.6_Fe_2.4_O_4_) CS structure where *T*_N_ < *T*_C_. Three samples with different core sizes (AFM core diameters 2, 5, and 16 nm) and constant FiM shell thickness (∼2 nm) were studied. All samples present strong magnetic coupling between the AFM and FiM materials. However, while the magnetic structure of each component is independent of the final particle size, the reduced dimension of the system forces the appearance of a variable superparamagnetic transition in the FiM shell region that is placed above or below the *T*_N_ of the AFM, depending on the particle size. The presence of this transition in the FiM shell significantly influences the exchange-coupled behavior of the system for small nanoparticles. Moreover, we speculate that for the smallest sample, another superparamagnetic transition is operating in the AFM material, which is closely correlated with the magnetic stability of the FiM counterpart. In essence, the size reduction of the NPs induces variations in the superparamagnetic behavior of both the AFM and the FiM components, ultimately impacting the overall magnetic properties of the CS NPs.

## Experimental Section

Monodisperse spherical NPs were synthesized through the thermal decomposition of a metal–oleate complex in a high-boiling solvent. The main details about the chemical synthesis can be found in ref. 22 Briefly, in a typical synthesis, 1.5 g of mixed metal–oleate complex (prepared previously)^22^ and 0.15 g of oleic acid were dissolved in 10 g of octadecene (Samples S1 and S2) or docosane (Sample S3) in a 50 mL three-neck round-bottom flask. The mixture was heated to the desired decomposition temperature of 3 °C min^–1^ for 2 h. Finally, the flask was removed from the heating mantle and allowed to cool down. During the heating, digestion, and cooling processes, the mixture was exposed to an N_2_ flow. All NPs were washed by several cycles of coagulation with ethanol, centrifugation at 5000 rpm, disposal of supernatant solution, and redispersion in hexane.

Low-resolution transmission electron microscopy (TEM) images were obtained using a CM12 Philips microscope with a LaB_6_ filament operated at 100 kV. High-Resolution TEM images were acquired with an image-corrected Titan3 (Thermo Fischer Scientific) Ultrahigh-Resolution Transmission Electron Microscope (UHRTEM) with a working voltage of 300 kV. The X-ray powder diffraction (XRD) patterns were recorded using a Bruker New D8 ADVANCE ECO diffractometer with Cu Kα radiation. Quantitative analysis was performed using the MAUD program.^67^ X-ray absorption spectroscopy (XAS) and X-ray magnetic circular dichroism (XMCD) experiments at the Fe and Co L_3_ edges were performed at the BOREAS beamline at the ALBA Synchrotron in Barcelona operating in transmission mode, at 5 K, under an applied magnetic field of 50 kOe. The NPs were dispersed in hexane and placed dropwise onto a carbon-supported grid, which was then attached to a copper sample holder. XAS and XMCD spectra were calculated within the Ligand Field Multiplet (LFM) model using the CTM4XAS 5.0 program,^68^ including spin–orbit coupling, crystal field effects, and reduction of the Slater integrals to include the interatomic configuration interaction. The Slater integrals were reduced by multiplying by factors of 80%, and the crystal field parameters were 1.5 eV (0.6 eV) for Co in *O_h_* (*T*_*d*_) sites and 0.6 eV (0 eV) for Fe in *O_h_* (*T*_*d*_) sites. The magnetic properties of the NPs were measured on tightly packed powdered samples using a vibrating sample mode magnetometer with 120 kOe (MagLab VSM12T-Oxford) and 90 kOe (VSM, Quantum Design PPMS) maximum field. Magnetization versus temperature measurements were performed in zero-field cooled (ZFC) and field-cooled (FC) conditions with 50 Oe or 30 kOe probe fields. Hysteresis loops were measured in ZFC and FC conditions after cooling from RT to 10 K with a 120 kOe applied field.

## Results and Discussion

Samples S1, S2, and S3 with 6(1), 9(1), and 20(2) nm mean diameter have been synthesized through a one-pot thermal decomposition of a mixed Fe^3+^-Co^2+^ oleate precursor (see Scheme 1 and Figure 1a). Details of the synthesis and the structural characterization are described in a previous work^22^ and in Scheme 1. Briefly, the NPs are composed of a cobalt–iron monoxide AFM core and a cobalt ferrite FiM shell of the formula Co_0.3_Fe_0.7_O|Co_0.6_Fe_2.4_O_4_. The chemical composition and structure of the core and shell regions are the same for all the samples, as well as the shell thickness, which is ∼2 nm independently of the particle diameter.^22^ The CS architecture is well distinguished also in the high-resolution transmission electron microscopy (HRTEM) image of Sample S3 (Figure 1b); moreover, the fast Fourier transform (FFT) analysis of the core and shell regions confirms the presence of rock-salt (Co_0.3_Fe_0.7_O) and cubic spinel structures (Co_0.6_Fe_2.6_O_4_), respectively (Figures 1b and S1).

**Scheme 1:**
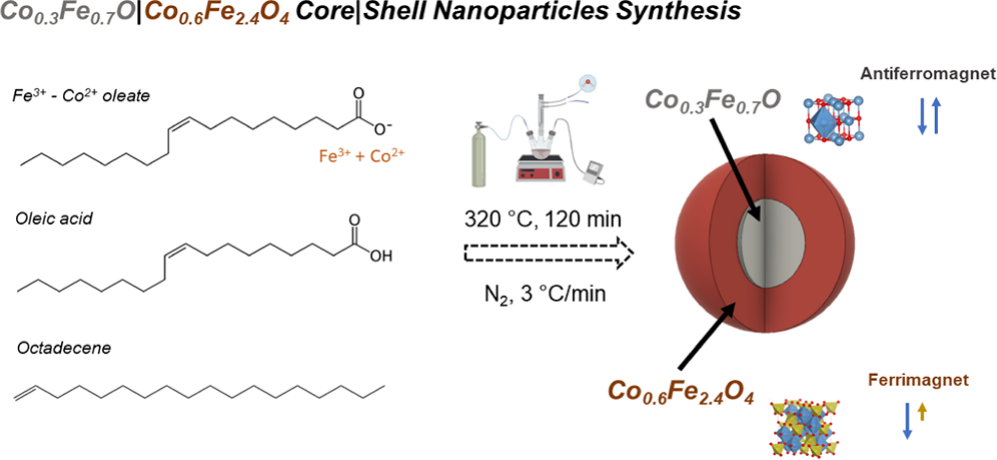
Schematic Representation of the Co_0.3_Fe_0.7_O|Co_0.6_Fe_2.7_O_4_ Core|Shell Nanoparticle Synthesis

**Figure 1:**
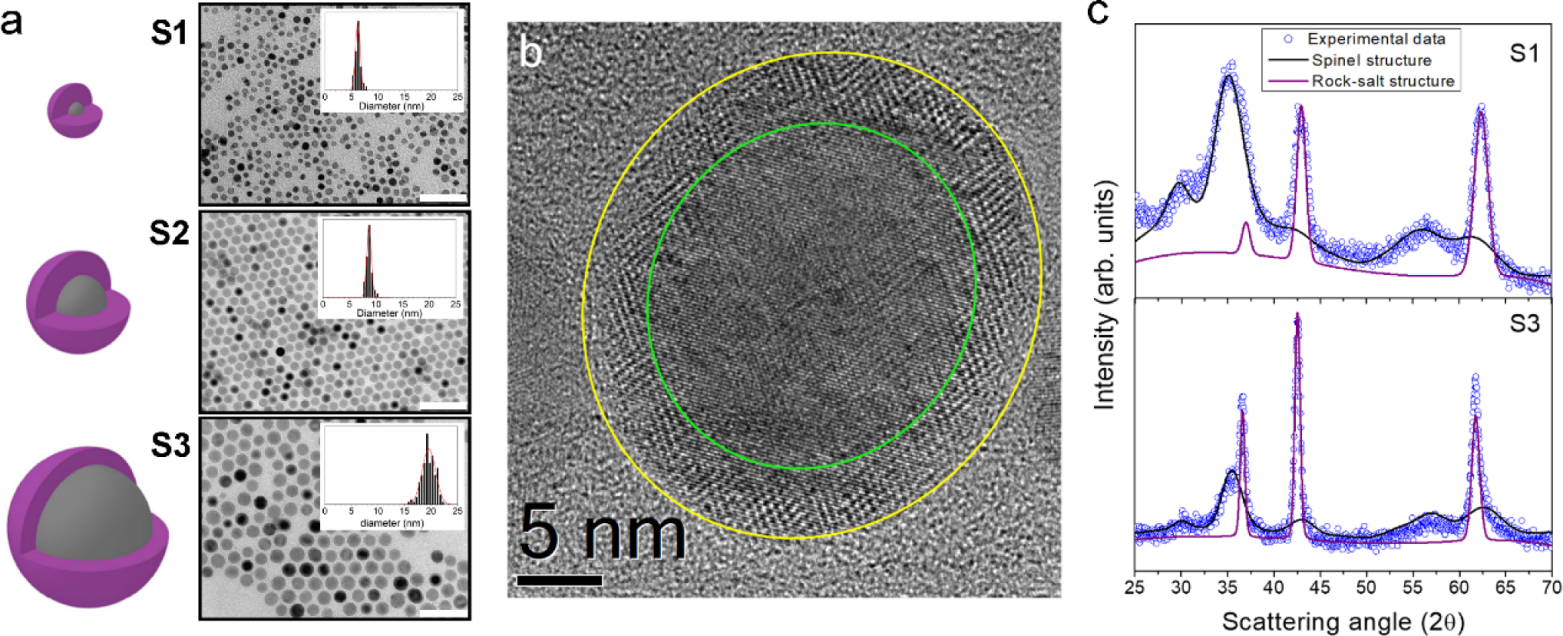
(a) Schematic representation of the bimagnetic CS NPs (lilac and gray colors refer to Co_0.6_Fe_2.4_O_4_ spinel shell and Co_0.3_Fe_0.7_O rock-salt core regions, respectively) and the corresponding TEM images and particle size histograms for Samples S1, S2, and S3 (white scale bars correspond to 50 nm). (b) HRTEM image for Sample S3 where the green and yellow circles depict the core and shell region, respectively. (c) Experimental and computed X-ray diffraction (XRD) patterns for Samples S1 and S3 with the specific contribution of the core and the shell region to the total computed fit.

The formation of the bimagnetic structure has been described to occur through the initial reduction of the precursor Fe^3+^ ions to Fe^2+^ in the synthetic media, leading to the formation of rock-salt metal-monoxide structure, i.e., Co_1–*x*_Fe_*x*_O, with the cationic ratio depending on the initial Fe/Co content of the oleate precursor.^23^ The exposure to noninert conditions causes the partial oxidization of iron ions to Fe^3+^, forming a stable, passivating shell layer of spinel ferrite Co_*x*_Fe_3–*x*_O_4_ by a mediated diffusion and growth mechanism, where the oxygen deposition onto the rock-salt surface creates a potential gradient from the surface to the inner core.^24–26^ The X-ray diffraction (XRD) analysis (Figure 1c) and the corresponding Rietveld refinement (Table S1) corroborate the formation of the bimagnetic structure, with crystal sizes comparable to those previously observed in the TEM analysis.

To further investigate the composition and magnetic structure of the CS NPs, X-ray absorption (XAS) and X-ray magnetic circular dichroism (XMCD) spectra were measured in transmission mode at low temperatures under high field conditions. To quantify the Fe and Co cation distribution over the tetrahedral (*Td*) and octahedral (*Oh*) sites, the experimental XAS and XMCD spectra of each sample were fitted using a linear combination of the Ligand Field Multiplet (LFM) model calculated spectra, using parameters close to those described in ref. 27,28 (see the Materials and Methods section). Figure 2a,b depict the XAS and XMCD experimental data at the Fe and Co L_3_-edges for Samples S2 and S3. The XMCD spectra are very close to that expected for a cobalt ferrite structure, as corroborated by comparing the spectra with those obtained for the reference sample, ***S***_**CFO**_, made up of 7 nm pure Co_0.6_Fe_2.4_O_4_ NPs (the synthesis, the morpho-structural, and magnetic properties of the sample can be found in Figure S2 and Table S2 and in ref 29). Conversely, XAS spectra show a marked difference when comparing XAS spectra of S2 and S3 and the reference **S**_**CFO**_. Such apparent discrepancy reflects the inherent difference of the two techniques: XAS is sensitive to the overall NP (i.e., core and shell) while XMCD measures the magnetic contribution from the FiM shell only. Tables S3 and S4 present the calculated Fe and Co percentages of occupancy calculated from XMCD and XAS data. The net increase in the amount of Co^2+^-*Oh* ions in the XAS spectra for Sample S2 reveals that the satellite Co^2+^-*Td* and Co^3+^-*Oh* cations are mainly placed at the shell region, as expected from the growth mechanism of the cobalt ferrite shell.^22^ Similar conclusions can be reached comparing XAS/XMCD spectra of the Fe^3+^ at *Td* sites. XMCD and XAS results are in close agreement with previous magnetometric analysis,^22^ revealing the AFM and FiM magnetic ordering of the core and shell regions, respectively.

**Figure 2:**
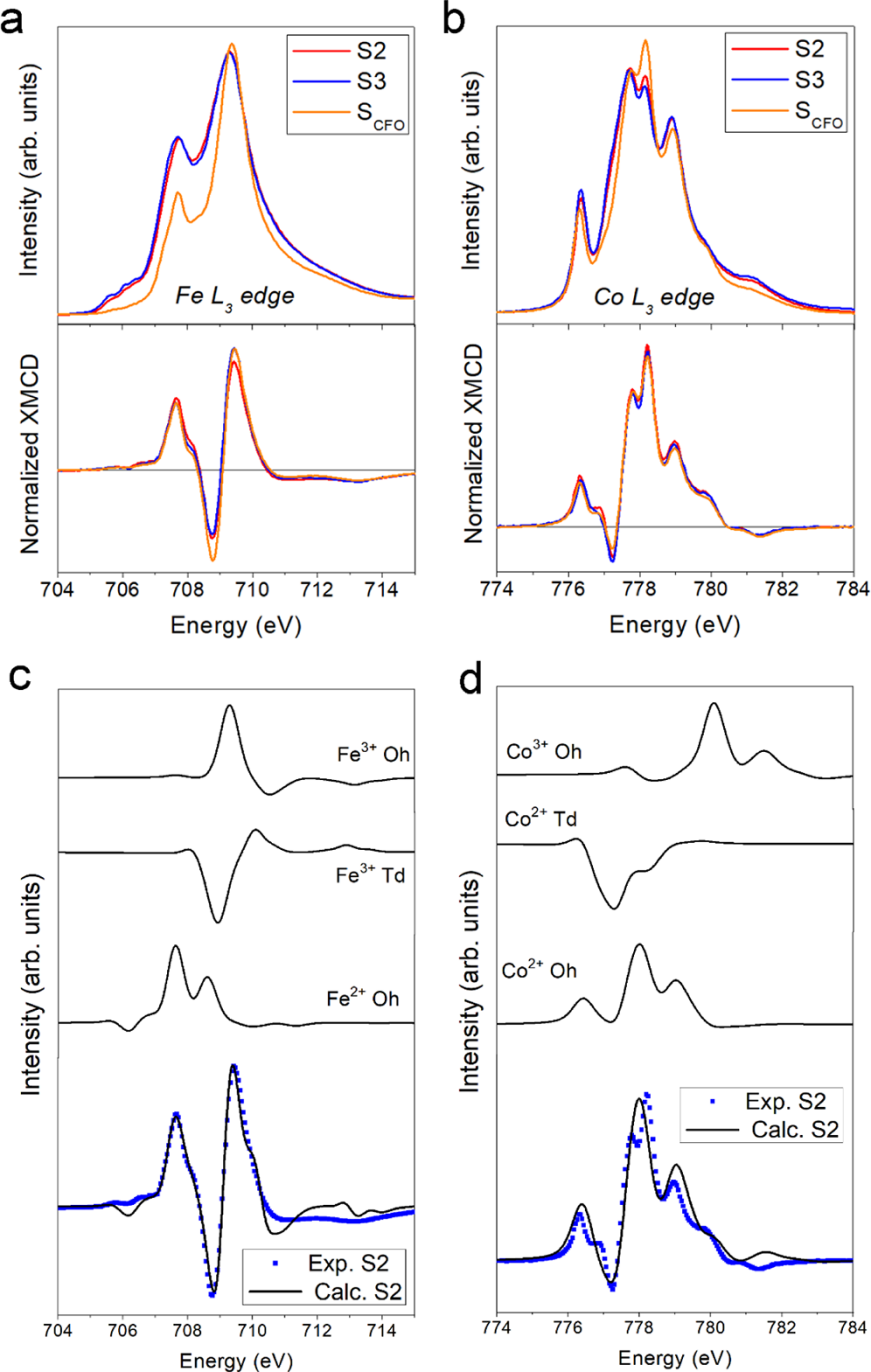
Experimental XAS and XMCD spectra at the (a) Fe and (b) Co L_3_ edge for CS Samples S2 and S3 and reference **S**_**CFO**_ and comparison between experimental and calculated XMCD spectra at the (c) Fe and (d) Co L_3_ edge for Sample S2.

Figure 3a presents the low-field magnetization vs temperature, *M*(*T*), curves recorded in zero field cool (ZFC) and field cool (FC) conditions. The curves are dominated by the presence of the characteristic superparamagnetic blocking temperature (*T*_B_) of the FiM shell. This temperature scales linearly with the shell volume, indicating that the magnetic anisotropy of the FiM counterpart remains practically independent of the particle size.^21^ Conversely, the AFM nature of the core region is revealed by the presence of a small peak at 220 K in Sample S3, while for S2, a similar peak can be observed only when the *M*(*T*) curve is recorded at a higher field (3 T). In both cases, the peak is located at a similar temperature, as expected for a thermodynamic transition^30^ and, thus, can be ascribed to the Néel temperature (*T*_N_) of the Co–Fe monoxide core region.^31^ For the smallest sample, this peak is not observed, probably due to the low AFM contribution to the total magnetization.^32^ On varying the core size, the CS system displays a different relative order of the blocking temperature of the FiM component, *T*_B_, and of the ordering temperature of the AFM phase, *T*_N_: for Sample S3, *T*_B,S3_ > 350 K is larger than *T*_N_; for Sample S2, *T*_B_ decreases due to the reduced size, and *T*_B,S2_ ∼ 200 K ∼ *T*_N_; for the smallest sample, although *T*_N_ cannot be discerned in the magnetization curves, it can be safely assumed that it is well above *T*_B_, and *T*_B,S1_ ∼ 110 K < *T*_N_. Note that the volume reduction of the AFM core is expected to force a small decrease in its value.^33^

**Figure 3:**
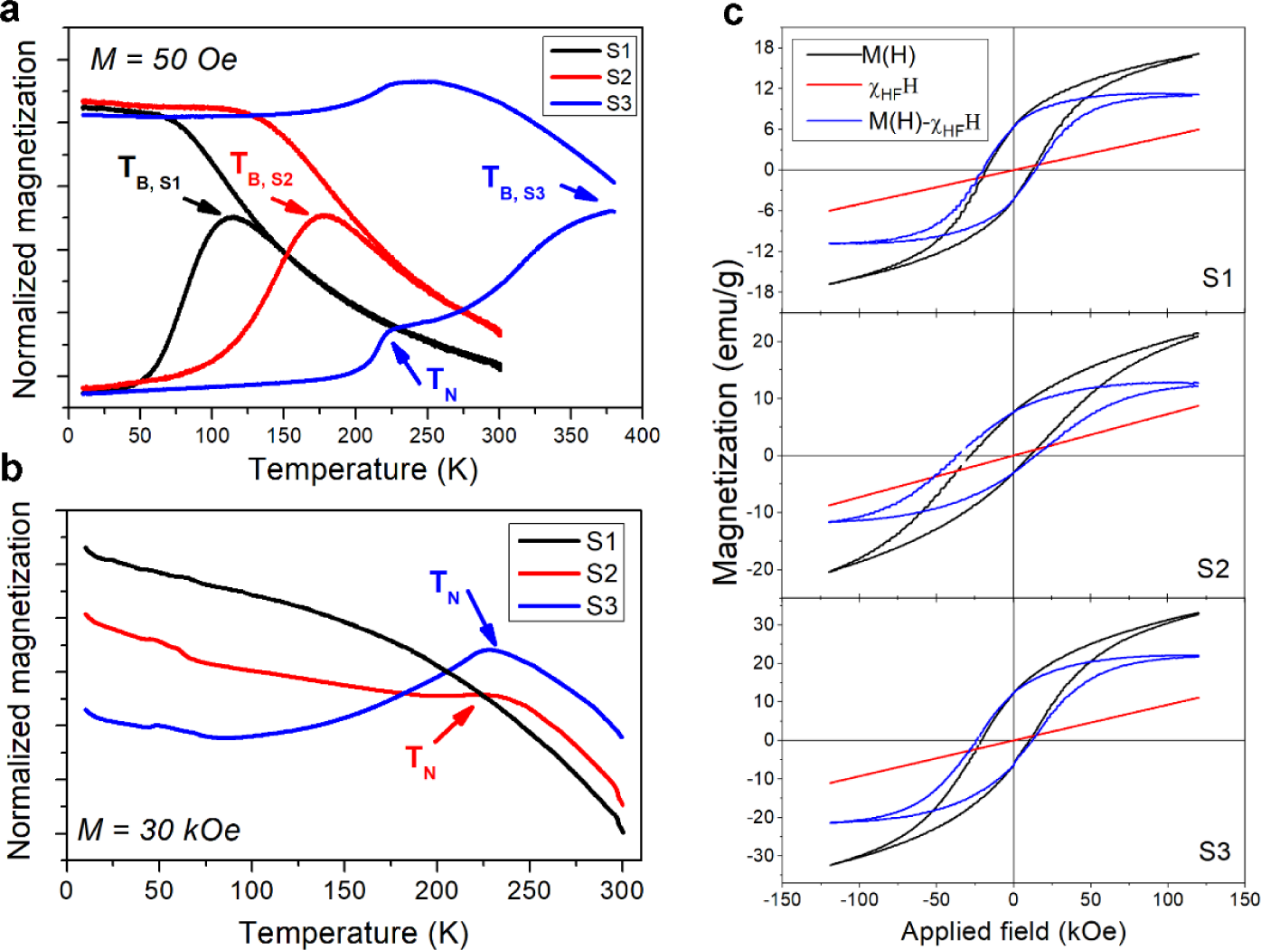
Magnetization versus temperature curves measured under ZFC-FC (a) and FC (b) protocols, respectively, at (a) low and (b) high applied magnetic field for Samples S1, S2, and S3. (c) FC hysteresis loops for the three samples (black curves), high-field susceptibility, χ_HF_, and corrected *M*_FM_ magnetization, *M*(*H*) = *M*_FiM_(*H*) + χ_HF_(*H*).

The low temperature (10 K) magnetization vs applied field curve, *M*(*H*), recorded after ZFC procedure depicts the classical hysteretic behavior for blocked NPs with large coercive fields (*H*_C_), and the corresponding large magnetic irreversibility (*H*_I_), the overlapping point between ascending and descending branches. Moreover, differently from classical hysteresis loops, we found pronounced high field slope. The hysteresis curves recorded under FC conditions (Figure 3c) depict similar characteristics, but the loop is horizontally shifted and has a coercive field larger by ca. 30% (see Figure S3). The major contribution to the high *H*_C_ and irreversible field of the samples is driven by the cobalt ferrite shell. On the other hand, the exchange coupling between the FiM shell layer and the AFM core region induced under FC entails an additional contribution to the magnetic anisotropy of the system responsible for the shift and of the increase of *H*_C_.^14^ Even if similar large *H*_C_ have been obtained for single-phase cobalt ferrite NPs,^34,35^ the large *H*_I_ is characteristic of our NPs only and arises from the combination of size and morphological effects.^22^ On the other hand, the nonsaturation behavior of the magnetization observed in both FC and ZFC curves can be explained by the magnetic contribution of the high anisotropic AFM core. It should be noted that although the FC and ZFC hysteresis loops are well far from the saturation regime (minor loop condition), no vertical shifts were observed.^22^

To better describe the magnetic behavior of the CS system, the hysteresis loops were analyzed by considering two different magnetic contributions, where the total magnetization is defined as *M*(*H*) = *M*_FiM_(*H*) + χ_HF_(*H*). Here *M*_FiM_ is the magnetization due to FiM shell and χ_HF_ is the high-field susceptibility determined by linear fitting the high field data (>70 kOe, see Figure 3b). In the first approximation, χ_HF_ can be ascribed to the AFM counterpart in the CS NP;^36,37^ indeed, although similar nonsaturation behaviors have been previously reported for hollow structures,^38^ exchange-coupled systems,^36^ spin-glass states,^39,40^ and canted spins in the FiM region,^41,42^ in all these cases, the value of the high field susceptibility was far below that observed here. To corroborate its nature, χ_HF_ per AFM mass unit was estimated at increasing temperatures from 5 K to *T*_B_, and the obtained data are reported in Figure 4. It can be observed that the χ_HF_ temperature dependence resembles the behavior expected for bulk AFM polycrystalline susceptibility,^43^ with a nearly constant value from 0 to 150 K and with a broad maximum at *T*_N_. The independent variation of χ_HF_ for *T* ≤ *T*_N_ allows to rule out the spin-glass state origin of the nonsaturation behavior.^44^ In addition, χ_HF_ decreases with the diameter of the AFM core: the largest sample, S3, exhibited susceptibility values close to those reported in the literature for single-crystal CoO^45,46^ (ca. 58 emu·Oe^1–^·g_AFM_^–1^), while much smaller χ_HF_ values were observed for S2 (ca. 14 emu·Oe^1–^·g_AFM_^–1^) and S1 (0.35 emu·Oe^1–^·g_AFM_^–1^). This decrease can be interpreted as an increase in the AFM core’s anisotropy and is probably related to surface or interface effects,^14,47,48^ as previously observed in AFM MnO NPs.^33,47,49^ This behavior supports the conclusion that χ_HF_ originates from the AFM core region of the CS NPs. Within this assumption, the FC and ZFC hysteresis loops can be corrected to extract only the FiM contribution, i.e., *M*_FiM_(*H*). The as obtained curves are shown in Figure 3c. First, it can be observed that all the samples saturate at fields in the range of 50 kOe, in close agreement with the behavior observed for single-phase cobalt ferrite particles.^29,34^ On the other hand, the calculated saturation magnetization, *M*_S_, of the samples (*M*_S,S1_ = 11 emu/g, *M*_S,S2_ = 16 emu/g, and *M*_S,S3_ = 45 emu/g), although rescaled to the amount of the FiM phase only (being the volume ratio *V*_FiM_/*V*_AFM_ 26, 7, and 1 for Samples S1, S2 and S3, respectively), are extremely low in comparison with cobalt ferrite in bulk^50^ or NP^29^ form. It can be assumed that this pronounced reduction comes out from the specific morphology and the spin configuration of the FiM counterpart.^40,51,52^ In fact, although single domain structure should be expected (the single domain size of the bulk Cobalt ferrite is around 25 nm^29^), the FiM shell is characterized by a strong local surface anisotropy in which strength and directions vary between the three samples due to the change of curvature radium. Considering that bulk exchange length (related to the spin exchange and not to the magnetic anisotropy) is around 5 nm, we can argue that a competition between the number of coherent domains (that increase with the size) and their random orientation with the decrease of the surface anisotropy (as the particle size increases) is operating, which may originate different magnetic incoherent reversal processes that, at the end, determine different *H*_C0_ values for the three samples.

**Figure 4:**
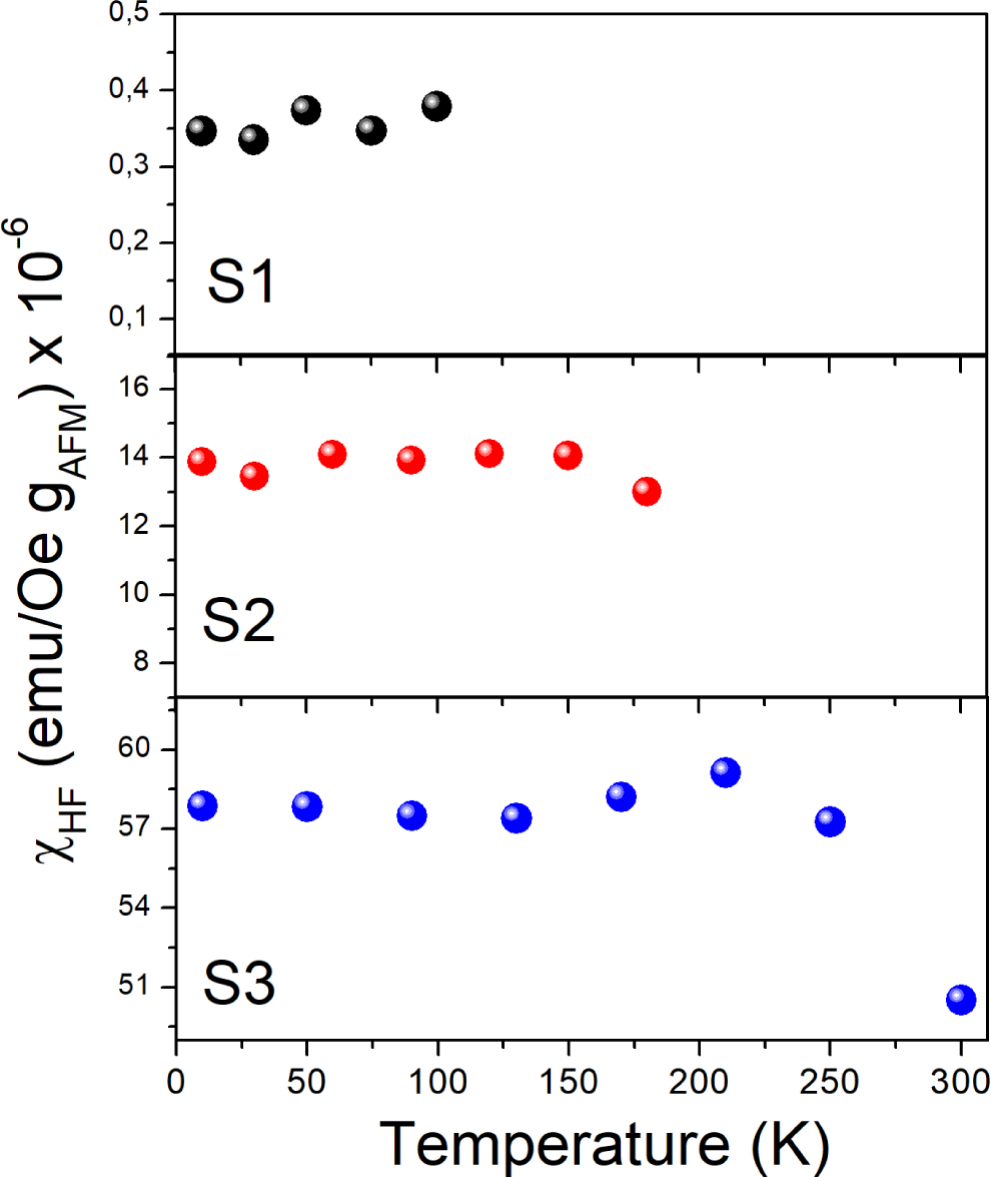
Temperature dependence of the high-field susceptibility χ_HF_ per mass unit of the AFM core material for Samples S1, S2, and S3.

Finally, to unravel the role played by the relative position between *T*_B_ and *T*_N_ in the exchange-coupled system, we studied the temperature dependence of *H*_C_ and *H*_E_ obtained from the FC loops. To this aim, the samples were cooled down to 10 K in the 50 kOe field, and then the loops were measured at progressively higher temperatures (Figure S5). The obtained data are reported in Figure 5a. A different trend as a function of particle size was observed: for the largest sample, *T*_B,S3_ > *T*_N_, both parameters decrease monotonically as the temperature increases; however, while *H*_E_ disappears at *T*_N_, *H*_C_ is still visible above this transition, as it has been indeed extensively reported for AFM-FM­(FiM) exchange-coupled systems with *T*_C_ > *T*_N_.^53^ Conversely, for Samples S1 and S2, both *H*_C_ and *H*_E_ disappear at the same temperature, i.e., at the blocking temperature of the FiM material; moreover, for S1, *H*_E_ displays a much faster decay at lower temperatures. In Figure S4, the normalized *H*_E_ values are reported as a function of the scaling parameter *T*/*T*_B_. For all the samples, two different regimes can be recognized: a strong decay at lower temperature followed by a monotonous decrease until *T* ∼ *T*_B_, where *H*_E_ vanishes. While the gradual decline for *H*_C_ and *H*_E_ is linked to the intrinsic thermal dependence of the magnetic anisotropy of each component,^54^ the strong decline observed for *H*_E_ in Sample S1 can be ascribed to training effects occurring in the AFM material, resulting in a decrease of *H*_C_ and *H*_E_ upon successive magnetic loops. Typically, training effects are noted in AFM-FM exchange-coupled structures or spin-glass systems,^14,55^ and they have two different origins: between the first and second loop (10 and 20 K in our experiment), where the effect is much more pronounced, the origin is associated with the magnetic symmetry and/or microstructure of the AFM material;^14,56^ for the following cycles (higher temperatures in the experiment), it is linked to the relaxation of uncompensated spins at the interface.^57^ However, taking into account that no spin-glass transition is observed either in the *M*(T)^32,58^ or χ_HF_(T)^44^ curves, we can hypothesize that the large training effects observed in Sample S1 could come from the reduced size of the AFM core region and/or the large FiM-to-AFM volume ratio.^14,59–61^

**Figure 5:**
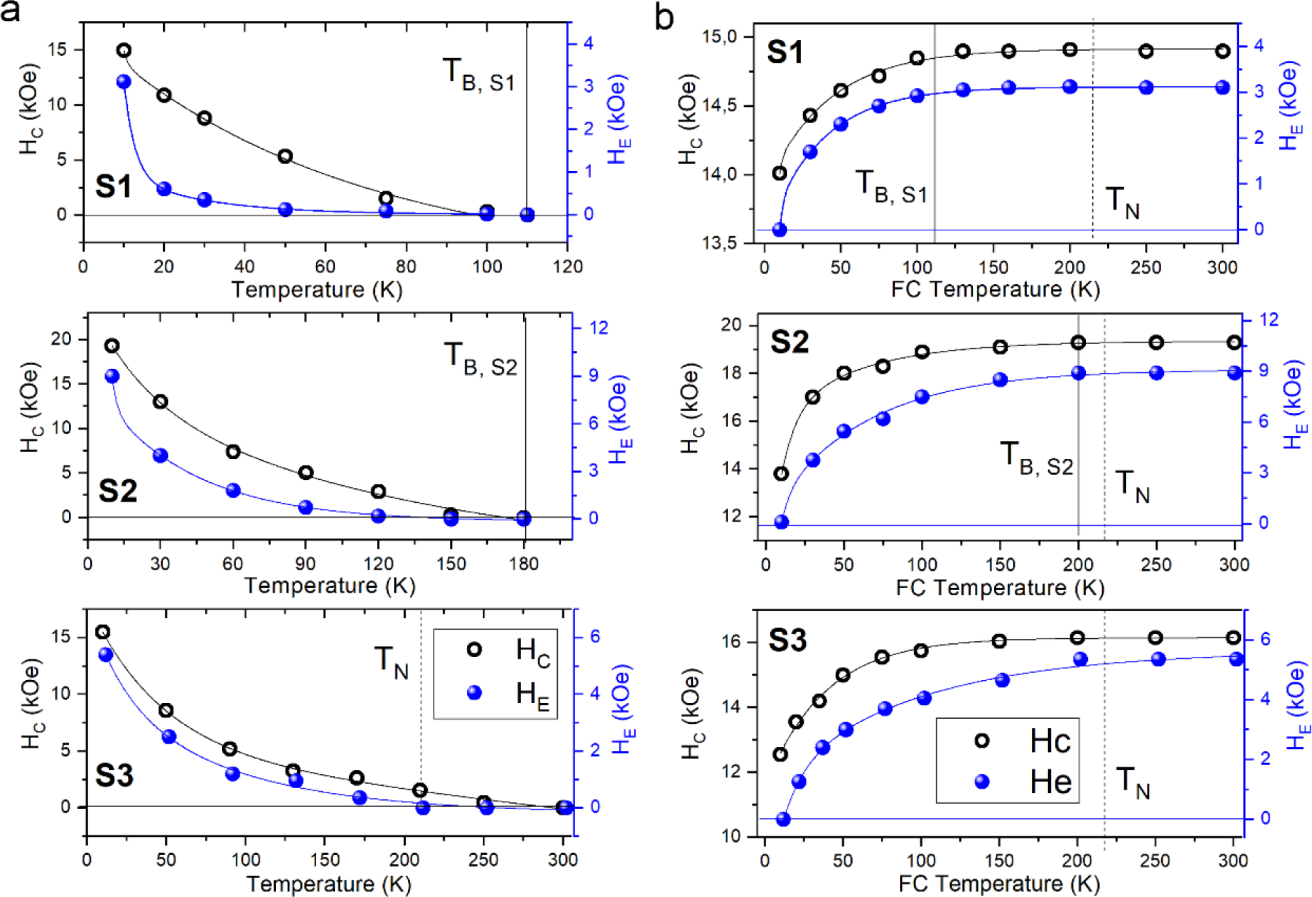
Coercive field, *H*_C_, and horizontal loop shift, *H*_E_, recorded (a) at increasing temperatures after FC to the lowest measuring temperatures (10 K) and (b) recorded at 10 K after applying the cooling magnetic field at variable temperatures, for Samples S1, S2, and S3. The solid lines are guide for the eyes. The hysteresis loops from which *H*_C_ and *H*_E_ values were estimated are provided in Figure S5.

For understanding the thermal dependence of *H*_C_ and *H*_E_, a series of hysteresis loops were measured by applying the FC field at variable temperatures. In Figures 5b and S5 the *H*_C_ and *H*_E_ values measured at 10 K as a function of the starting temperature of the FC process are reported. It is well-known that the horizontal hysteresis loop is a characteristic of the interface exchange coupling between AFM and FM­(FiM) materials in close contact, i.e., exchange bias.^17^ This effect is observed while cooling the system, usually with a *T*_C_ > *T*_N_, with a static magnetic field applied at *T*_C_ > *T* > *T*_N_.^17^ Sample S3 displays the behavior expected for NPs, which satisfy this condition: both *H*_C_ and *H*_E_ are independent of the cooling temperature for *T* > *T*_N_ and then they decrease monotonically for lower *T*, confirming the presence of the AFM material which causes the additional anisotropy.^17^ Similar behavior is depicted by Sample S2: both parameters start decreasing when the cooling temperature is 200 K, which is in the ranges of the AFM *T*_N_ and of the FiM *T*_B,S2_. Conversely, for the smallest sample, the trend is completely different, since in this case, *H*_C_ and *H*_E_ remain almost constant when the field is applied down to *T*_B,S1_ = 110 K and then they start decreasing below *T*_B,S1_. This behavior agrees with the disappearance of *H*_E_ at *T*_B,S1_ < *T*_N_ due to the thermal fluctuations in the shell region, forcing the closing up of the hysteresis loop at *T*_B,S1_ (Figure 5a).^14^ However, the dependence observed for *H*_E_ in Figure 5b cannot be explained in the same way. For magnetic coupling to occur between a FiM and an AFM material, the FiM must be ordered when crossing the *T*_N_ of the AFM.^14^ To achieve this, a magnetic field is applied (FC procedure), which first aligns the magnetic moments of the FiM and, subsequently, upon crossing *T*_N_, those of the AFM (systems with *T*_C_ > *T*_N_).

However, Sample S1 deviates from this scheme, since *H*_E_ and *H*_C_ can be observed even when the field is applied at *T* < *T*_N_.^33,49,61^ A possible explanation to this discrepancy involves finite-size effects in the AFM counterpart, with thermal fluctuations in the core region being responsible for the appearance of an AFM blocking temperature, *T*_B,S1‑AFM_.^14^ The presence of *T*_B_ in AFM materials can be experimentally verified by measuring the disappearance of the hysteresis loop shift^62^ or analyzing the *M*(*T*) curves,^32,63^ among others.^64,65^ However, since in our case no clear feature of the AFM blocking transition can be observed in the low or high field ZFC-FC curves (see Figure 3), the presence of a *T*_B,AFM_ can only be inferred indirectly by observing the influence of the AFM core on the magnetic properties of the FiM material. Based on these considerations, we can hypothesize that the size reduction of the core region forces the presence of a blocked–unblocked transition in the AFM material. Notably, data reported in Figure 5b show that for S1, the decrease in *H*_E_ occurs at the same temperature as *T*_B,S1_. However, it cannot be ruled out that such a superparamagnetic-blocked state transition in the AFM material may occur at much lower values due to the significantly reduced dimensionality of the core but are increased by the magnetic proximity effect between the two magnetic layers.^30,65^ We stress that the possibility that the observed behavior in S1 arises from a low temperature shift of the ordering temperature well below 220 K of the AFM component can be discarded. Indeed, although the *T*_N_ dependence with size has been already reported in AFM NPs,^33^ these variations are usually small and tend to increase *T*_N_ as the NP size decreases.^33,49,66^

## Conclusions

In conclusion, here we presented an investigation on a family of exchange-coupled AFM|FiM Co_0.3_Fe_0.7_O|Co_0.6_Fe_2.4_O_4_ CS NPs with a variable core-to-shell volume ratio. We found that, unlike the magnetic structure, which is practically independent of the particle size, the magnetic properties are strongly dominated by the presence of the AFM material and by the size of the two components. The three samples exhibit different high-field susceptibility and shifted relative position of the blocking temperature of the FiM shell and the Néel temperature of the AFM core. For larger NPs, the system can be described as a classic *T*_C_ (*T*_B,FiM_) > *T*_N_ exchange-coupled system in which the exchange-bias effect is activated by the magnetic ordering of the AFM core; however, as the particle size is reduced, the *T*_B,FiM_ decreases, reaching for the smallest sample a value well below the *T*_N_ of the AFM. In this case, the exchange-bias is activated by the reduction of the relaxation-time (thermal activated fluctuations) of the FiM shell. Our results suggest that in that temperature range, the AFM material undergoes a blocked–unblocked transition, *T*_B,AFM_, that activates the magnetic coupling between both nanometric components. Establishing optimal operating conditions is essential for designing future devices exploiting exchange bias effects, as the phenomenon is significantly influenced by the size of the ferrimagnetic and antiferromagnetic components. The interplay between these components is thus key to defining performance parameters and stability in advanced applications.

## Supplementary Material



## Notes

Fe and Co percentage occupancies obtained from calculated XAS and XMCD spectra for samples S_2_, S_3_, and S_CFO_; HRTEM image and FFT analysis for sample S_3_; TEM image and particle size histogram for reference sample S_CFO_; Experimental and calculated XMCD spectra for sample S_2_ and reference S_CFO_; Comparison of *H*_E_ dependence as a function of temperature for samples S_1_, S_2_, and S_3_ (PDF)
