# Supplementary material for: Competitive Size Effects in Antiferromagnetic|Ferrimagnetic Core|Shell Nanoparticles for Large Exchange Bias
Source: ACS Appl Nano Mater. 2024 Dec 4;7(23):27489–97. doi: 10.1021/acsanm.4c05505 (PMC12584292; doi:10.1021/acsanm.4c05505)
Supplement: Supplementary file 1 [file an4c05505_si_001.pdf]

Supporting information

Competitive size effects in Antiferromagnetic |Ferrimagnetic Core|Shell Nanoparticles for large Exchange Bias

Alberto López-Ortega[1,2*], Beatrice Muzzi[3,5*], Cesar de Julián Fernández[4], Claudio Sangregorio[3,5]

lopezortega.alberto@gmail.com; beatrice.muzzi@iccom.cnr.it

[1]*Departamento de Ciencias, Universidad Pública de Navarra, E-31006 Pamplona, Spain.*
[2]*Institute for Advanced Materials and Mathematics (INAMAT[2]), Universidad Pública de Navarra, E-31006 Pamplona, Spain.*
[3]*ICCOM - CNR, I-50019 Sesto Fiorentino (FI), Italy.*
[4]*IMEM - CNR, I-43124 Parma, Italy.*
[5]*Dept. of Chemistry "U. Schiff", University of Florence and INSTM, I-50019 Sesto Fiorentino (FI), Italy*

Table S1. Summary of the structural data obtained from XRD analysis for samples **S1**, **S2** and **S3**.[a]

Sample	Spinel structure		Rock salt structure	
	Cell parameter (nm)	Crystal size (nm)	Cell parameter (nm)	Crystal size (nm)
**S1**	0.8420 (1)	2.3 (5)	4.200 (1)	6.3 (5)
**S2**	0.8413 (1)	2.1 (5)	4.247 (1)	7.4 (5)
**S3**	0.8411 (1)	3.0 (5)	4.254 (1)	14.8 (5)

[a]Lattice parameter and crystal size are obtained by Rietveld refinement of the experimental patterns. Uncertainties on the last digit are given in parentheses.

Table S2. Summary of the compositional, morphological, structural and magnetic data of sample **S_{CFO}**.[a]

Sample	Compositional, morphological and structural data				Magnetic data			
	Stoichiometry	TEM	XRD		10K			T_B (K)
		Particle size (nm)	Crystal size (nm)	Cell parameter (nm)	M_{5T} (emu/g)	M_R (emu/g)	H_C (kOe)	
S_{CFO}	$Co_{0.6}Fe_{2.4}O_4$	7(1)	8.8(5)	0.840(1)	77.4	58.8	13.5	220

[a]The determination of cobalt and iron concentrations was performed by x-ray fluorescence spectrometry. Lattice parameter and crystal size are obtained by Rietveld refinement of the experimental patterns. Uncertainties on the last digit are given in parentheses. The errors for M_{5T}, M_R, H_C and T_B have been assessed to be the 5 and 2%, respectively, of the experimental values.

Table S3. Fe and Co percentage occupancy obtained from calculated XMCD spectra for samples **S2**, **S3** and **S_{CFO}**.

Sample	Fe			Co		
	Fe^{2+} Oh	Fe^{3+} Td	Fe^{3+} Oh	Co^{2+} Td	Co^{2+} Oh	Co^{3+} Td
**S2**	28	38	34	10	86	4
**S3**	30	37	33	10	86	4
**S_{CFO}**	28	38	34	9	86	5

Table S4. Fe and Co percentage occupancy obtained from calculated XAS spectra for samples **S2**, **S3** and **S_{CFO}**.

Sample	Fe			Co		
	Fe^{2+} Oh	Fe^{3+} Td	Fe^{3+} Oh	Co^{2+} Td	Co^{2+} Oh	Co^{3+} Td
**S2**	48	26	26	5	93	2
**S3**	50	24	26	5	93	2
**S_{CFO}**	31	34	35	6	90	4

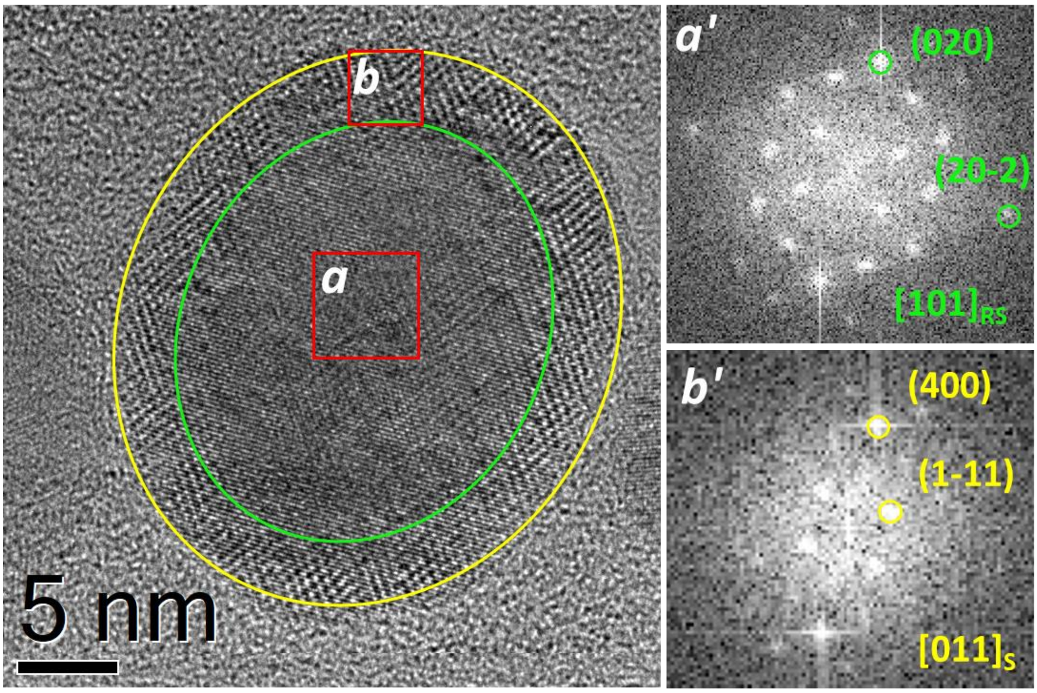

Figure S1. Left side: HRTEM image of an **S3** NP, where 2 regions (red squares *a* and *b*) are selected: *a* and *b* correspond to $Co_{0.3}Fe_{0.7}O$ rock-salt core and $Co_{0.6}Fe_{2.4}O_4$ spinel ferrite shell, respectively. Right side: The FFT analysis of the two regions showing the presence of different crystallographic structures; the labelled spots are related to crystallographic planes that can be indexed as (*a'*) rock-salt phase ($Fm\bar{3}m$), in zone axis $[101]_{RS}$ and (*b'*) cubic Spinel structure ($Fd\bar{3}m$), $[011]_S$.

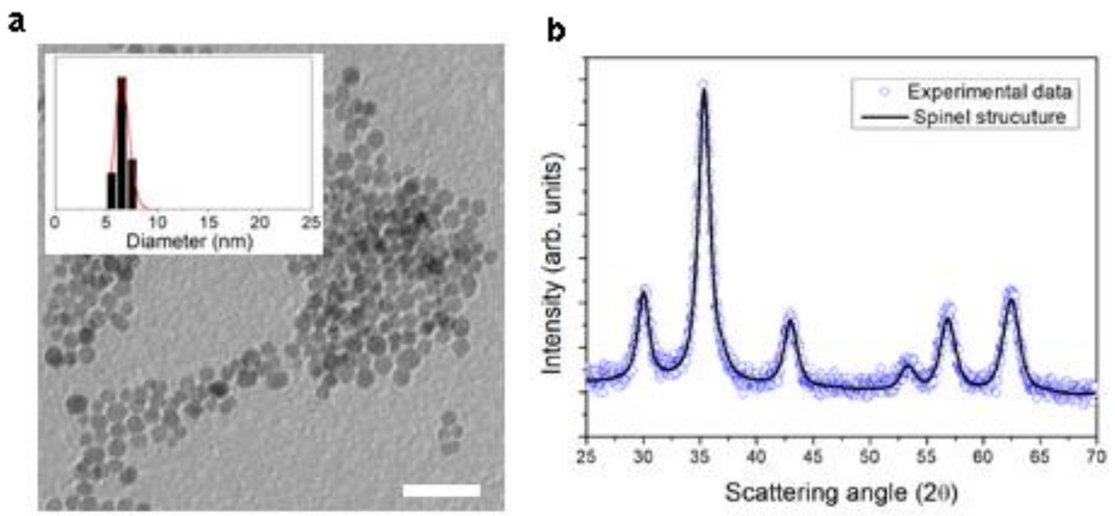

Figure S2. TEM image and the corresponding particle size histograms (white scale bar corresponding to 50 nm) and (b) experimental and computed x-ray diffraction pattern patterns for reference sample $\mathbf{S_{CFO}}$.

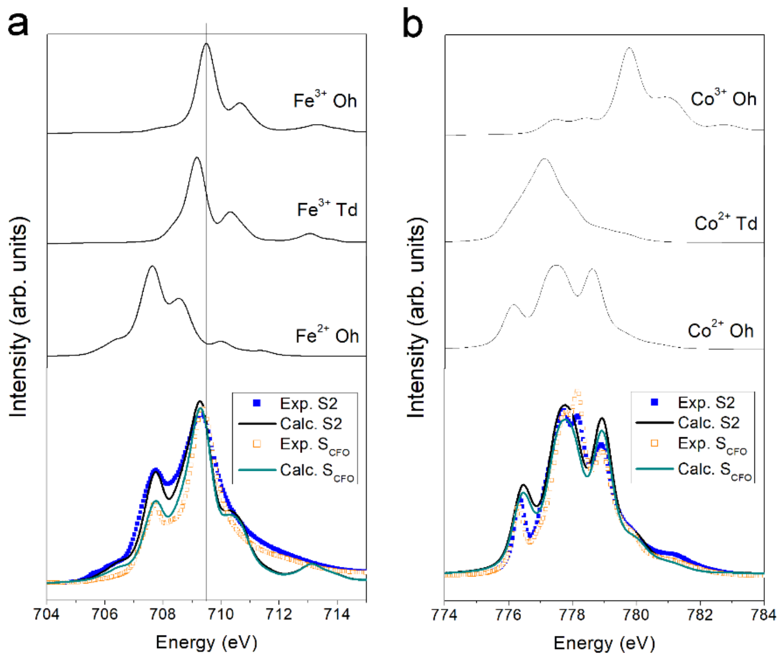

Figure S3. Experimental and calculated XAS spectra at the (a) Fe and (b) Co L_3 edge for samples **S2** and reference **S_{CFO}**.

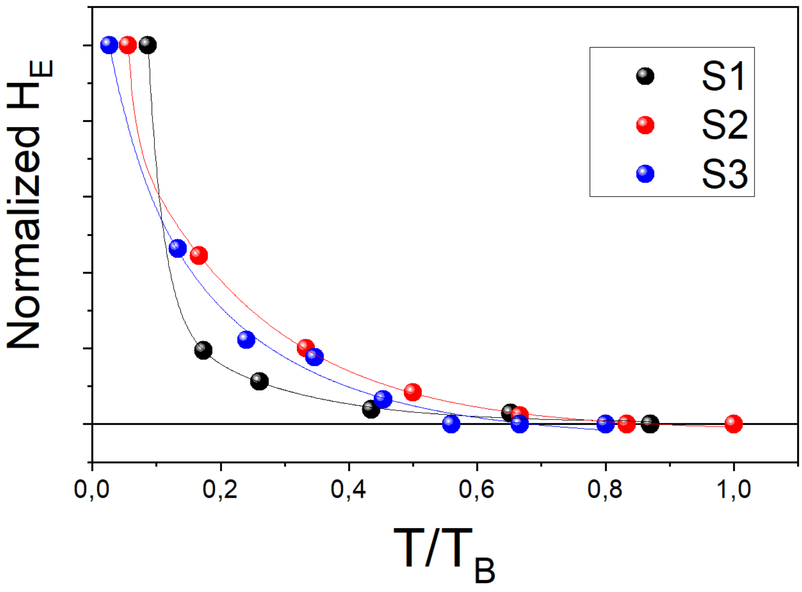

Figure S4. Comparison of the normalized horizontal loop shift, H_E, as a function of the scaled temperature T/T_B for samples **S1**, **S2** and **S3**. The solid lines are guide for the eyes.

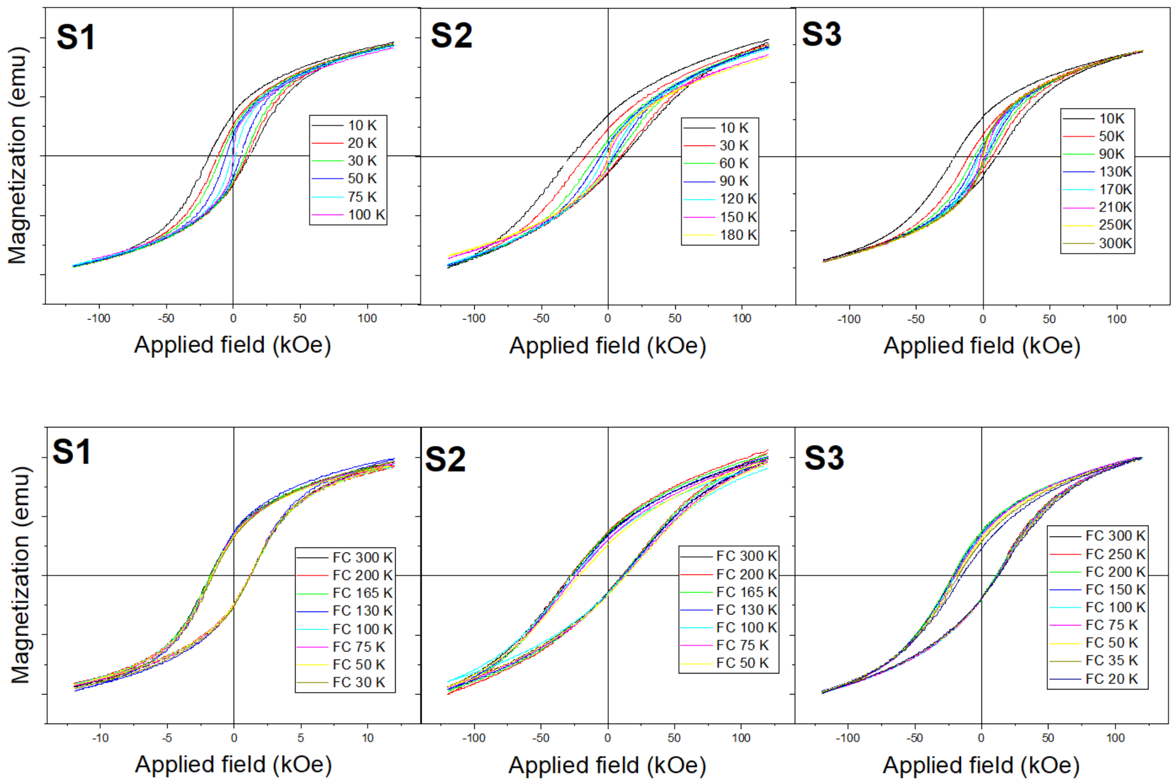

Figure S5. Magnetization versus applied field curves for samples S1, S2 and S3. The upper row displays the hysteresis loops measured at different temperatures after field cooling, while the lower row shows the low-temperature hysteresis loops after field cooling at decreasing temperatures. The H_C and H_E values estimated from the loops are reported in Figure 5a (upper row loops) and Figure 5b (lower row loops), respectively.
